# Drought Analysis for Kuwait Using Standardized Precipitation Index

**DOI:** 10.1155/2014/451841

**Published:** 2014-10-20

**Authors:** Jaber Almedeij

**Affiliations:** Civil Engineering Department, Kuwait University, P.O. Box 5969, 13060 Safat, Kuwait

## Abstract

Implementation of adequate measures to assess and monitor droughts is recognized as a major matter challenging researchers involved in water resources management. The objective of this study is to assess the hydrologic drought characteristics from the historical rainfall records of Kuwait with arid environment by employing the criterion of Standardized Precipitation Index (SPI). A wide range of monthly total precipitation data from January 1967 to December 2009 is used for the assessment. The computation of the SPI series is performed for intermediate- and long-time scales of 3, 6, 12, and 24 months. The drought severity and duration are also estimated. The bivariate probability distribution for these two drought characteristics is constructed by using Clayton copula. It has been shown that the drought SPI series for the time scales examined have no systematic trend component but a seasonal pattern related to rainfall data. The results are used to perform univariate and bivariate frequency analyses for the drought events. The study will help evaluating the risk of future droughts in the region, assessing their consequences on economy, environment, and society, and adopting measures for mitigating the effect of droughts.

## 1. Introduction

Drought is a phenomenon, which may affect areas located in wet or dry environments, resulting in insufficient moisture caused by a deficit in precipitation over some time period [1]. A thorough review of drought definitions was provided by Wilhite and Glantz [2] who classified drought into six overall categories of meteorological, climatological, atmospheric, agricultural, and hydrologic and water management aspects. The time scale over which precipitation deficits accumulate is important to highlight these classifications. For example, agricultural droughts have typically a short-time scale of one month when soil moisture and rainfall are inadequate to support crop growth leading to the loss of yield, while hydrologic droughts have intermediate- and long-time scales of 3, 6, and 12 months or higher with marked depletion of surface and subsurface water such as lakes, streams, reservoirs, and groundwater [3, 4]. The reduced surface and subsurface water amounts due to hydrologic droughts increase the risk of water shortage, especially when the water demand increases in all major use sectors due to growth in population and economic activities.

Many indices were developed to assess and monitor drought characteristics quantitatively. Among them is the Palmer Drought Index [5], which is based on the concept of water balance. The computation of this index involves calibrating parameters including precipitation, evapotranspiration, runoff, and soil moisture. This index is applied within the United States but has little acceptance elsewhere [6]. One explanation for this is provided by Guttman [7] and Ray and Shewale [8], who suggested that in humid areas this index represents more of agricultural drought, whereas in semiarid and arid areas it represents hydrologic drought.

Another widely acceptable index based on probability concept is the Standardized Precipitation Index (SPI) [1, 9]. Precipitation anomalies are a naturally recurring feature of global climate [10], affecting various components of the hydrologic cycle to produce a drought. This index, which considers only precipitation for its computation, provides a better representation of abnormal wetness and dryness than the Palmer Index [11]. SPI represents the difference of precipitation from the mean divided by the standard deviation, where these two statistical parameters are determined from past continuous records, ideally of at least 30 years ago [1]. Owing to the reason that this index is standardized, it can be used to assess the drought impact worldwide (e.g., [4, 12–14]). For a given location, the SPI may also be computed for any time scale whether short, intermediate, or long by simply estimating the probability distribution function for the time scale selected. This will be useful to address the impact of the different drought categories mentioned earlier.

The effects of drought often accumulate over time. Two important drought characteristics widely used in the literature to assess the cumulative effect are severity and duration (e.g., [15, 16]). Drought severity is defined as the cumulative deviation for SPI values below a threshold level, while the time period when this occurs is termed as the drought duration (e.g., [17]). The threshold level for drought severity can be taken as a constant SPI value or a function varying with time [18]. Both drought characteristics of severity and duration are correlated variates, where different combination values of them may generate quite different drought effects. Therefore, for drought risk assessment, it is useful to construct a joint probability distribution from these two variates and perform frequency analysis.

The aim of this research is to investigate droughts observed in the historical rainfall records of Kuwait using the Standardized Precipitation Index criterion. The computation of SPI values will consider the time scales of 3, 6, 12, and 24 months. This will be useful for intermediate- and long-term assessments of hydrologic droughts affecting, for example, groundwater recharge ability in the country and increasing the risk of water shortage. A short-term scale assessment of one month is not relevant here due to the desert environment of Kuwait, by which the country does not rely on rainfall to support agricultural surfaces, and rather it depends on nonconventional water resources such as seawater desalination and wastewater treatment and reuse. The drought characteristics of both severity and duration will also be estimated in this study. The 3-month SPI scale will be used as an example of employing these two drought characteristics to perform bivariate frequency analysis.

## 2. Case Study

The climate of Kuwait is of arid environment, where rainstorms are infrequent with short duration but torrential. The average depth of annual evaporation is high approaching a value of 4000 mm, while the annual depth of rainfall is low varying from 35 mm to 242 mm. Temperature during summer (winter) reaches an average daily high temperature of 43°C (15°C), with the average daily low temperature falling to 23°C (5°C). Summer temperatures can be even higher when hot winds blow from the desert. Winter temperatures can be classified as mild but occasionally become cold when northerly or northwesterly winds bring cold air from the north.

The arid environment of Kuwait causes a water shortage problem. Essentially, the only existing conventional water resource is fresh groundwater with relatively limited quantities. The limited groundwater quantities are due to the few areas of actual surface water runoff and accumulation as evaporation always exceeds available precipitation. Fresh groundwater is found in the depressions of Rawdatain and Umm Al-Aish, which are located in the northern area of Kuwait [19]. The freshwater of Rawdatain is kept as a reserve and some amount is marketed as bottled mineral water, while the water of Umm Al-Aish has been contaminated from massive crude oil spillage by the retreating Iraqi army during the 1990 Gulf War [20]. Nonconventional water resources have become important in helping overcome the existing water shortage problem in the country. Two alternatives have been employed, which are seawater desalination and wastewater treatment and reuse. However, the water production cost is considered relatively high for these two alternatives, and the seawater desalination which relies on multistage flash suffers from environmental issues [21].

**Figure 1:**
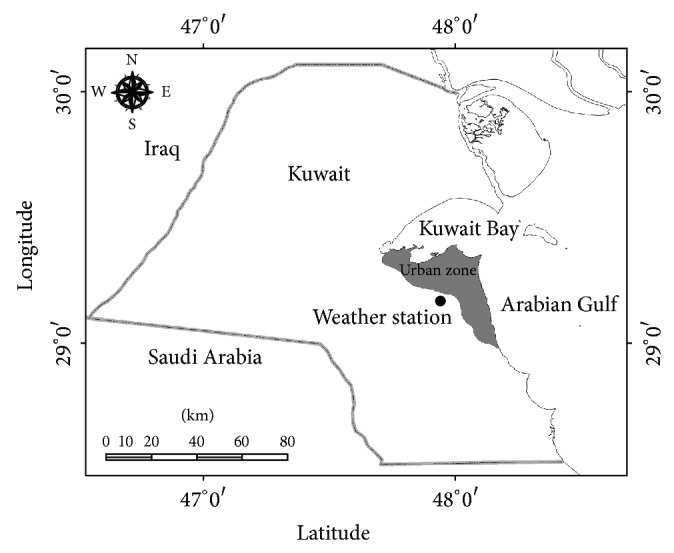
Location of the weather station.

**Figure 2:**
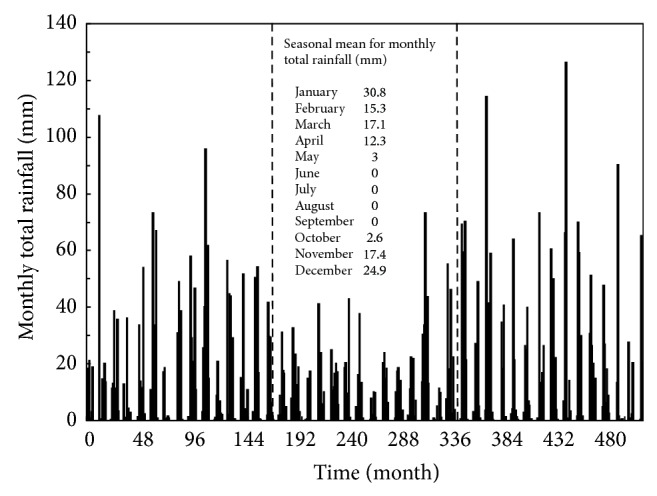
Rainfall data available from the weather station located at Kuwait International Airport for the time duration from January 1967 (month number 1) to December 2009 (month number 516). The two dashed lines are used to divide the rainfall data into three distinct equal time intervals, at April 1981 (month number 172) and August 1995 (month number 344).

The monthly total rainfall data of Kuwait will be used in this study to perform the drought analysis, which would help monitoring the fresh groundwater available. Owing to the relatively small area of Kuwait, which is about 18,000 km^2^, rainfall data collected for a point estimate can be considered spatially representative [22]. Accordingly, monthly total rainfall data readily available from the weather station located in Kuwait International Airport can be employed for the analysis (Figure 1). The rainfall data is plotted in Figure 2 for the time duration from January 1967 to December 2009, with 516 monthly observations. This is the widest range of rainfall records available in Kuwait at the weather station. It should be mentioned that the data measurements from August 1990 to June 1991 were not recorded by the weather station because of the Iraqi invasion of Kuwait. To maintain continuity in terms of time, this lack of information has been handled here by averaging the data by considering the seasonal mean resulting from adding the value of the same month but for the year before and after and then dividing by two.

## 3. SPI Calculation and Results

The SPI is equivalent to the *Z*-score often used in statistics. However, for a series of rainfall measurements with a time scale of 12 months or less, the distribution of the data is usually considered skewed. Thom [23] found that the gamma distribution fits the rainfall data more appropriately. The probability density function for the gamma distribution *g*(*x*) is defined as follows:

(1) $$\begin{array}{c} g\left(x\right)=\frac{1}{\beta \Gamma \left(\alpha \right)}x^{\alpha -1}e^{-x/\beta }, \end{array}$$

where *α* > 0 is shape parameter, *β* > 0 is scale parameter, and *x* is rainfall measurement. The gamma function Γ(*α*) in the above equation is defined as

(2) $$\begin{array}{c} \Gamma \left(\alpha \right)=\overset{\infty }{\underset{0}{\int }}y^{\alpha -1}e^{-y}dy. \end{array}$$

Fitting the gamma distribution to the rainfall data requires estimating *α* and *β*. Edwards and McKee [24] suggested estimating these parameters by using the approximation of Thom [23] for maximum likelihood to obtain

(3) α^=14A(1+1+4A3),β^=x−α^,

where

(4) $$\begin{array}{c} A=\ln\left(\overset{-}{x}\right)-\frac{\sum \ln\left(x\right)}{n}. \end{array}$$

  *n* is number of rainfall measurements, and $\overset{-}{x}$ is the mean of *x*.

Integrating *g*(*x*) with respect to *x* and inserting the estimates of *α* and *β* yield the expression for the cumulative distribution *G*(*x*) for a given month and time scale:

(5) $$\begin{array}{c} G\left(x\right)=\overset{x}{\underset{0}{\int }}g\left(x\right)dx=\frac{1}{{\hat{\beta }}^{\hat{\alpha }}\Gamma \left(\hat{\alpha }\right)}\overset{x}{\underset{0}{\int }}x^{\hat{\alpha }-1}e^{-x/\hat{\beta }}dx. \end{array}$$

Assuming that $t=x/\hat{\beta }$, this cumulative distribution becomes

(6) $$\begin{array}{c} G\left(x\right)=\frac{1}{\Gamma \left(\hat{\alpha }\right)}\overset{x}{\underset{0}{\int }}t^{\hat{\alpha }-1}e^{-t}dt. \end{array}$$

Since the gamma function is undefined for *x* = 0 and the rainfall data may contain zero measurements, the cumulative distribution may be conveniently expressed as

(7) $$\begin{array}{c} H\left(x\right)=q-\left(1-q\right)G\left(x\right), \end{array}$$

where *q* is the probability of a zero. That is, if *m* is the number of zero measurements in a rainfall time series, Thom [23] states that *q* can be estimated by *m*/*n*. The cumulative distribution *H*(*x*) is then transformed into the standard normal random variable *Z* by employing the approximate conversion provided by Abramowitz and Stegun [25] as

(8) Z=SPI=−(t−c0+c1t+c2t21+d1t+d2t2+d3t3) for  0<H(x)≤0.5,Z=SPI=+(t−c0+c1t+c2t21+d1t+d2t2+d3t3) for  0.5<H(x)<1.0,

where

(9) t=ln⁡⁡(1H(x)2) for  0<H(x)≤0.5,t=ln⁡⁡(11.0−H(x)2) for  0.5<H(x)<1.0.

The coefficients in (8) are equal to *c*_0_ = 2.515517, *c*_1_ = 0.802853, *c*_2_ = 0.010328, *d*_1_ = 1.432788, *d*_2_ = 0.189269, and *d*_3_ = 0.001308.

**Figure 3:**
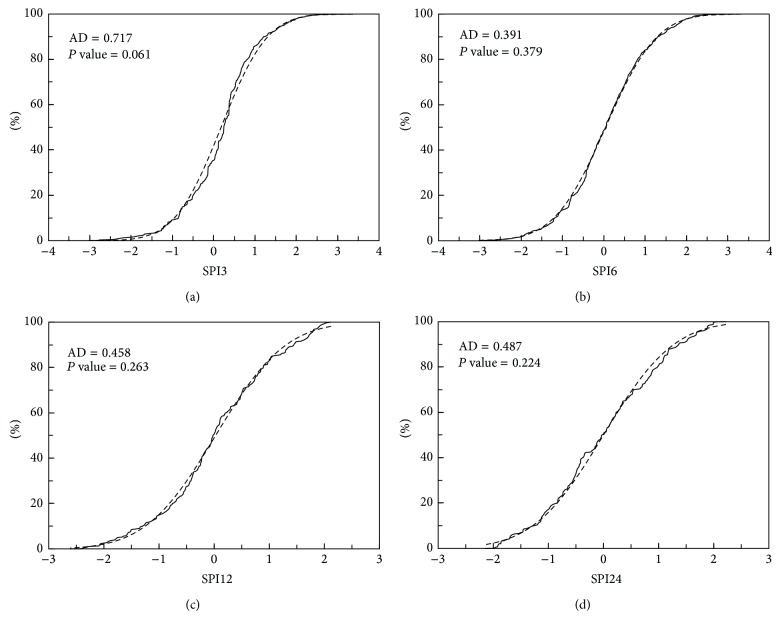
Standardized Precipitation Index (SPI) for the rainfall data plotted as probability distribution functions for the time scales of 3, 6, 12, and 24 months. The dashed curve represents the theoretical cumulative distribution, and the solid curve is the fitted empirical cumulative distribution. AD corresponds to Anderson-Darling statistic.

The above criterion was used here to estimate the SPI values for the rainfall data of Kuwait.  Figure 3 shows the results presented as probability distribution functions for the time scales 3, 6, 12, and 24 months. Here, the SPI values are termed correspondingly as SPI3, SPI6, SPI12, and SPI24. It is seen that the probability distributions are very close to normal, verified by using the Anderson-Darling normality test resulting in small statistics by which the hypothesis of normality was not rejected for the *P* value at the 0.05 significance level. The SPI classifications with regard to dry and wet events and the percentage available in each category in the time scales selected for the data of Kuwait are shown in Table 1. The SPI values are divided here arbitrarily into categories ranging from extreme wet (relative to the mean and standard deviation of the data) to extreme drought. The percentage available in the theoretical standard normal distribution is also presented in the table for a comparison with the categories for the data of Kuwait.

**Figure 4:**
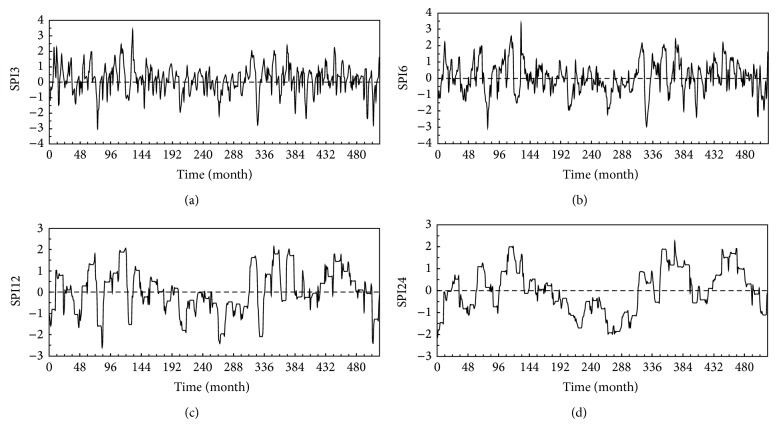
Standardized Precipitation Index (SPI) for the rainfall data plotted for the time scales of 3, 6, 12, and 24 months. The time duration of the data is from January 1967 (month number 1) to December 2009 (month number 516).

The temporal behavior of the SPI values is presented in Figure 4. On a small scale such as that for the SPI3 series, the drought intensities are highly variable and become less than −1.0 and greater than 1.0 on several occasions. This variation is due to a seasonal component found in the rainfall data. It is worth mentioning that the characteristics of the four seasons of spring, summer, autumn, and winter are not distinct in the arid environment of Kuwait, which can rather be classified into rainy and dry months. As it can be seen in Figure 2, the rainy months in Kuwait, on average, are November, December, January, February, March, and April. The drought patterns appear mainly after those months and become worse during summer, that is, June, July, and August. However, on a larger scale such as that for SPI24 series, the drought becomes less frequent and of longer duration. If the rainfall data in Figure 2 is divided into three distinct equal time intervals of 172 months, it can show that the interval in the middle has a rainfall amount lower than that of the others; the total rainfall amounts from the first to the third intervals are equal to 1870, 1420, and 2160 mm, respectively. One long-term drought that can be observed clearly in the SPI24 series has a total duration equal to about 10 years, occurring from month number 192 (December 1982) to 313 (January 1993). This drought event resulted obviously from the low rainfall amount of 1420 mm within the second time interval.

On the other hand, for all time scales shown in Figure 4, there is no long-term trend component recognized. This can be tested by fitting a linear regression trend to the observations resulting in slope and intercept values nearly equal to zero. Justifying the absence of a long-term trend of drought requires, however, testing a sufficiently wider historical rainfall data series, which is not available at this weather station. This justification would be useful to tell, for example, whether a phenomenon such as global warming affects somehow the severity or frequency of drought at this location.

**Figure 5:**
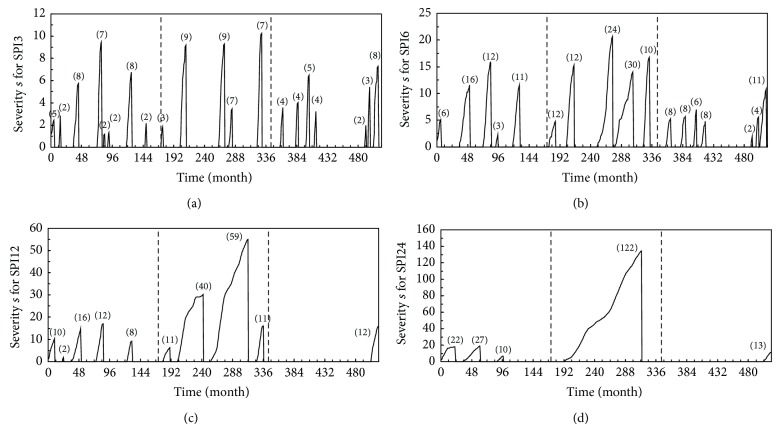
Drought severity for SPI3, SPI6, SPI12, and SPI24. The spikes represent the successful drought events. The numbers in the parentheses represent the drought durations. The time duration of the data is from January 1967 (month number 1) to December 2009 (month number 516). The two dashed lines are used to divide the data into three distinct equal time intervals corresponding to those found in Figure 2.

A drought event ends when the SPI value becomes positive. The drought severity is then the cumulative of SPI values within the drought duration. Figure 4 can be used to estimate the drought severity and duration. For convenience, the drought severity is taken to be positive as

(10) $$\begin{array}{c} s=-\overset{d}{\underset{i=1}{\sum }}{\text{SPI}}_{i}, \end{array}$$

where *s* is drought severity and *i* starts with the first month of a drought and continues until the end of the drought duration *d*. This relationship suggests that the longer the drought persists the worse the magnitude is [1]. As seen in Figure 5, the 3-month scale has the highest number of droughts among the other scales. While the 24-month scale has only five drought events, the magnitudes for these events are relatively large compared to those for the other scales. The worst drought event for this time scale is equal to *s* = 122, which is the one occurring from month number 192 to 313 mentioned earlier.

## 4. Frequency Analysis for Drought Events

To provide a comprehensive evaluation for droughts, one single variable such as drought severity is insufficient for the analysis. Instead, the bivariate characteristics of drought severity and duration can be used to derive a joint probability distribution. The drawback of bivariate distributions is the difficult mathematical derivations needed for fitting parameters from observed data. In recent years, copulas have been used for multivariate hydrological analysis to overcome such difficulties [26, 27]. A copula function offers great flexibility to select univariate distributions well fitted to observed data and construct a suitable multivariate distribution. It is worth noting that, in Figure 5, as the time scale is increased the number of spikes corresponding to successful drought events decreases. Accordingly, as the time scale is increased, it becomes less accurate to use severity-duration data points to fit a suitable probability distribution. However, to provide an example for fitting a joint probability distribution, the SPI3 series may be chosen here as it contains the largest number of severity-duration data points.

The Clayton copula can be used in this study to construct the bivariate distribution of drought severity and duration. The Clayton copula is an asymmetric Archimedean copula, which is considered appropriate in drought simulation because it is known to reflect tail structure of droughts well [15]. The Clayton copula function is given by

(11) $$\begin{array}{c} C_{\theta }\left(u,v\right)=\max\left[{\left(u^{-\theta }+v^{-\theta }-1\right)}^{-1/\theta },0\right], \end{array}$$

where the parameter *θ* is used to measure the degree of association between *u* and *v*. It is given by

(12) $$\begin{array}{c} \theta =\frac{2\tau }{1-\tau }, \end{array}$$

where *τ* is Kendall's tau. Typically, *θ* ∈ [−1, *∞*)∖{0}. For this study, 0 < *θ* < *∞* by which the above equation can be simplified to

(13) $$\begin{array}{c} C_{\theta }\left(u,v\right)={\left(u^{-\theta }+v^{-\theta }-1\right)}^{-1/\theta },\;\theta \in \left(0,\infty \right). \end{array}$$

The joint probability distribution for the univariate distributions of drought severity (*F*_*S*_) and duration (*F*_*D*_) can thus be expressed as

(14) $$\begin{array}{c} C_{\theta }\left(F_{S}\left(s\right),F_{D}\left(d\right)\right)={\left({F_{S}\left(s\right)}^{-\theta }+{F_{D}\left(d\right)}^{-\theta }-1\right)}^{-1/\theta },\;\theta >0. \end{array}$$

**Figure 6:**
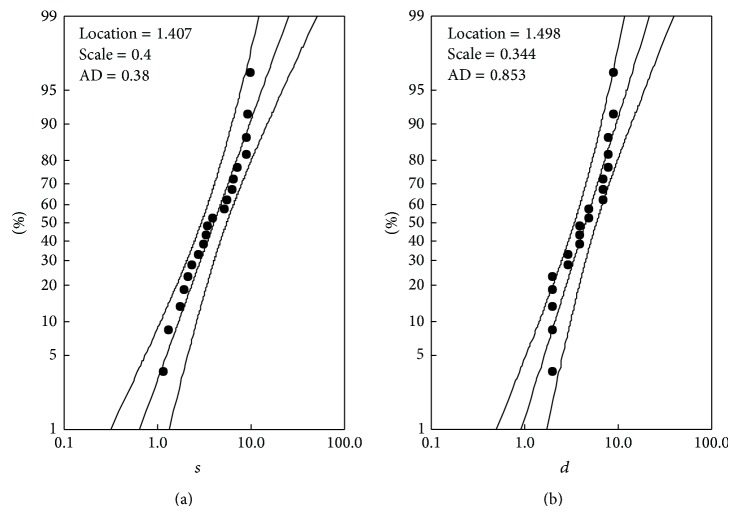
The probability plots for the log-logistic distribution of drought severity and duration for SPI3 series. The upper and lower solid curves represent the confidence intervals based on the location and scale parameters estimated from the data. AD corresponds to Anderson-Darling statistic.

The severity-duration data can then be used to determine the best fitting probability distribution functions for *F*_*S*_ and *F*_*D*_. The distributions most commonly used in the literature for such application are the gamma, Weibull, log-normal, and exponential. Among them, the log-normal distribution was found here with the best Anderson-Darling statistic. The cumulative log-normal distribution, however, needs a numeric approximation. Because the log-logistic distribution, which can be solved analytically, is similar to the log-normal, it can be used instead. The probability plots for the log-logistic distribution of drought severity and duration for the SPI3 series are shown in Figure 6. The log-logistic distribution is expressed as

(15) $$\begin{array}{c} F_{X}\left(x;\mu ;b\right)={\left[e^{-(\ln\;x-\mu )/b}+1\right]}^{-1}, \end{array}$$

where *μ* is location parameter, $b=\sqrt{3}\sigma$ is scale parameter, and *σ* is standard deviation. The values for the location and scale parameters for both functions *F*_*S*_ and *F*_*D*_ are shown in Figure 6. The probability plot shows also the associated confidence intervals based on the parameters estimated from the data.

**Figure 7:**
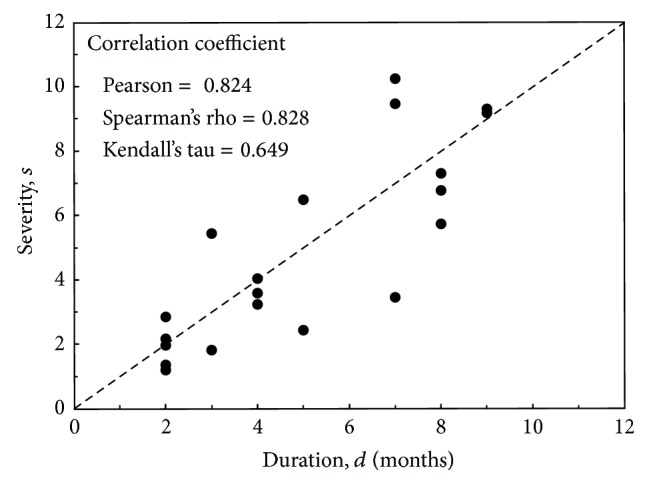
Drought severity versus duration for SPI3 series. The dashed line represents perfect agreement.

The bivariate correlation between drought severity and duration for the SPI3 series is shown in Figure 7. A positive correlation exists between the two drought variables. According to Pearson and Spearman's rho, the correlation is considered high, more than 0.8. Kendall's tau though is equal to *τ* = 0.649, which yields *θ* = 3.7 from (12). The copula becomes

(16) $$\begin{array}{c} C_{\theta }\left(F_{S}\left(s\right),F_{D}\left(d\right)\right)={\left({F_{S}\left(s\right)}^{-3.7}+{F_{D}\left(d\right)}^{-3.7}-1\right)}^{-1/3.7} \end{array}$$

with

(17) $$\begin{array}{c} F_{S}\left(s\right)={\left[e^{-(\ln s-1.407)/0.4}+1\right]}^{-1},\\ F_{D}\left(d\right)={\left[e^{-(\ln d-1.498)/0.344}+1\right]}^{-1}. \end{array}$$

**Figure 8:**
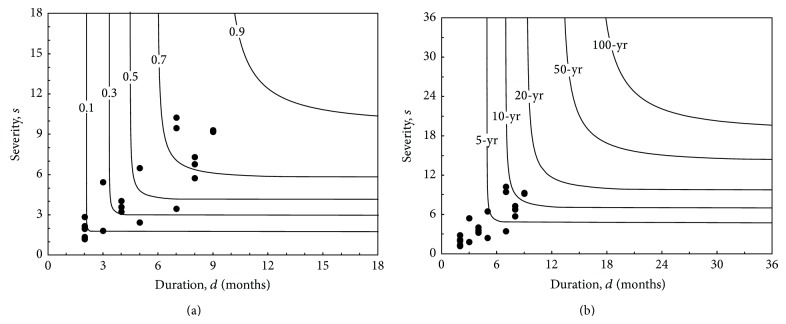
Contours for the two variates of drought severity and duration, with the solid circles representing the severity-duration data for SPI3 series: (a) joint probability distributions calculated by (16); (b) joint return periods calculated by (20).

Owing to the reason that various combinations of drought severity and duration can result with the same occurrence probability, the above equations are plotted as contour lines in Figure 8(a). This figure can be used to obtain the different combinations of drought severity and duration for a given occurrence probability.

The return period of drought events, defined as the average elapsed time between occurrences with a certain or greater magnitude, is traditionally calculated using a univariate distribution [28]. However, complex behaviors are usually characterized by a multivariate or bivariate distribution. For better assessment of drought, the distributions for both severity and duration can be used to obtain the bivariate return period. The univariate return period can also be estimated for this case to provide comparison. The single variable return period of drought in terms of year is defined for the partial drought severity *T*_*S*_ and partial drought duration *T*_*D*_ as

(18) $$\begin{array}{c} T_{S}=\frac{1}{\gamma P\left(S\geq s\right)}=\frac{1}{\gamma \left(1-F_{S}\left(s\right)\right)}, \end{array}$$

(19) $$\begin{array}{c} T_{D}=\frac{1}{\gamma P\left(D\geq d\right)}=\frac{1}{\gamma \left(1-F_{D}\left(d\right)\right)}, \end{array}$$

where *γ* is the average number of drought events per year [18, 29], calculated from Figure 5 for the SPI3 series as *γ* = 0.465. The bivariate return period of drought *T*_*SD*_ in terms of year can be derived as

(20) TSD=1γ(P(S≥s  or  D≥d))=1γ(1−FS,D(s,d))=1γ(1−Cθ(FS(s),FD(d))).

**Figure 9:**
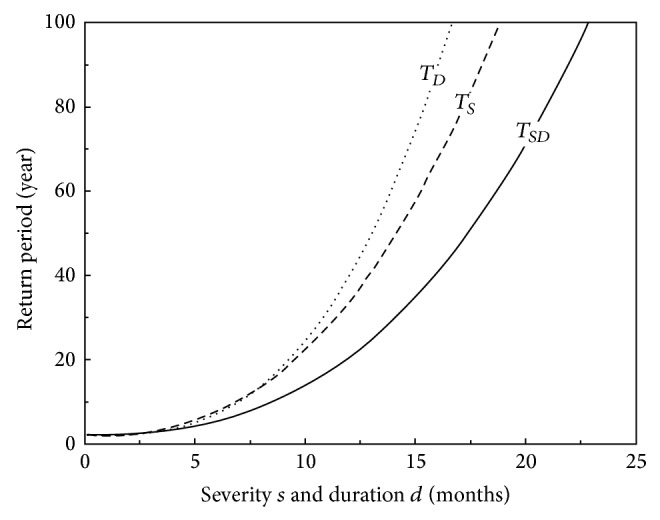
Univariate return period of severity *T*_*S*_ (18) and duration *T*_*D*_ (19) in terms of year plotted together with the bivariate severity and duration return period *T*_*SD*_ (20) in terms of year for SPI3 series.

The above three equations can be plotted together in Figure 9. Here, in order to plot *s* and *d* together in a single axis, it is assumed that they are identical in magnitude. This assumption is possible because of the nearly equivalent values observed in Figure 7. This is due to the fact that most of the SPI3 values are sufficiently close to −1.0, rendering *s* calculated by (10) very close in magnitude to *d*. It can be seen that, given a return period, *s* and *d* values obtained, respectively, by (18) and (19) are smaller than those obtained by (20). This implies that if one ignores the close correlation between *s* and *d* and employs a single variable return period, then the influence of drought event will be underestimated. The contours derived from (20) are plotted in Figure 8(b). The solid circles in the figure represent the severity-duration data points for SPI3 series. It is seen that the estimated bivariate return periods *T*_*SD*_ range from 2.3 to 11.6 years corresponding to occurrence probabilities from 0.065 to 0.815 shown in Figure 8(a).

## 5. Conclusions

This study assessed droughts observed in the desert environment of Kuwait by using the Standardized Precipitation Index criterion. It has been found for the given rainfall data that there is no long-term trend of drought, but only a seasonal variation pattern of time series component. Justifying the absence of the long-term trend requires though a sufficiently wider historical rainfall data series, which will be useful to examine whether a phenomenon such as global warming affects somehow the severity or frequency of drought at this location. This study also estimated the bivariate return period of the two severity and duration drought characteristics. A comparison presented in Figure 9 has shown that the bivariate return period is smaller than the univariate return period relying on either severity or duration. One may conclude accordingly that considering the bivariate frequency analysis for the assessment of drought data will help to avoid underestimating drought impact.

## Figures and Tables

**Table 1:** SPI values classification and the percentage available in the theoretical standard normal distribution and in the time scales selected for the data of Kuwait.

Class	SPI value^a^	Percentage in category (%)				
		SND^b^	3-month scale	6-month scale	12-month scale	24-month scale
Extreme wet	>2.0	2.28	1.94	2.13	0.78	0.39
Very wet	1.5 to 1.99	4.4	5.43	6.01	7.75	8.33
Moderate wet	1.0 to 1.49	9.19	6.78	7.75	8.72	10.27
Near normal	0.99 to −0.99	68.26	76.55	70.35	67.64	63.57
Moderate drought	−1.0 to −1.49	9.19	6.01	8.14	6.4	10.27
Severe drought	−1.5 to −1.99	4.4	1.55	3.68	6	6.98
Extreme drought	<−2.0	2.28	1.74	1.94	2.71	0.19

^a^SPI categories adopted from Bordi et al. [30].

^b^Standard normal distribution.
